# Fast Optical Signals for Real-Time Retinotopy and Brain Computer Interface

**DOI:** 10.3390/bioengineering10050553

**Published:** 2023-05-05

**Authors:** David Perpetuini, Mehmet Günal, Nicole Chiou, Sanmi Koyejo, Kyle Mathewson, Kathy A. Low, Monica Fabiani, Gabriele Gratton, Antonio Maria Chiarelli

**Affiliations:** 1 Department of Neuroscience, Imaging and Clinical Sciences, G. D’Annunzio University of Chieti-Pescara, 66100 Chieti, Italy; 2 Institute for Advanced Biomedical Technologies, G. D’Annunzio University of Chieti-Pescara, 66100 Chieti, Italy; 3 Beckman Institute, University of Illinois at Urbana Champaign, Urbana, IL 61801, USA; 4 Department of Computer Science, Stanford University, Stanford, CA 94305, USA; 5 Department of Psychology, Faculty of Science, University of Alberta, Edmonton, AB T6G 2R3, Canada; 6 Psychology Department, University of Illinois at Urbana Champaign, Champaign, IL 61820, USA

**Keywords:** fast optical signals (FOS), event-related optical signals (EROS), brain–computer interface (BCI), retinotopy, machine learning (ML)

## Abstract

A brain–computer interface (BCI) allows users to control external devices through brain activity. Portable neuroimaging techniques, such as near-infrared (NIR) imaging, are suitable for this goal. NIR imaging has been used to measure rapid changes in brain optical properties associated with neuronal activation, namely fast optical signals (FOS) with good spatiotemporal resolution. However, FOS have a low signal-to-noise ratio, limiting their BCI application. Here FOS were acquired with a frequency-domain optical system from the visual cortex during visual stimulation consisting of a rotating checkerboard wedge, flickering at 5 Hz. We used measures of photon count (Direct Current, DC light intensity) and time of flight (phase) at two NIR wavelengths (690 nm and 830 nm) combined with a machine learning approach for fast estimation of visual-field quadrant stimulation. The input features of a cross-validated support vector machine classifier were computed as the average modulus of the wavelet coherence between each channel and the average response among all channels in 512 ms time windows. An above chance performance was obtained when differentiating visual stimulation quadrants (left vs. right or top vs. bottom) with the best classification accuracy of ~63% (information transfer rate of ~6 bits/min) when classifying the superior and inferior stimulation quadrants using DC at 830 nm. The method is the first attempt to provide generalizable retinotopy classification relying on FOS, paving the way for the use of FOS in real-time BCI.

## 1. Introduction

Brain–computer interfaces (BCIs) are technologies that connect the central nervous system to a computer or another device [1,2]. BCIs can be used in humans for mapping brain functions, state monitoring, and enhancing or repairing cognitive and sensory–motor abilities. In contrast to conventional input devices such as a keyboard, pen, or mouse, BCI connects the human brain to peripheral devices by establishing a direct, interactive, and bidirectional link between the brain and the external environment [3,4]. A comprehensive BCI system typically consists of four components, a brain signal collector, an algorithm identifying and classifying incoming data, an algorithm transmitting decoded commands to the controlling equipment, and a device transmitting back feedback. Hence, the brain generates signals reflecting the user’s intentions, and the BCI system converts these signals into output commands for controlling external devices. In brain–machine fusion, BCI is an essential component of the exchange of information [4].

Portable and wearable neuroimaging techniques, such as electroencephalography (EEG) and functional near-infrared spectroscopy (fNIRS), are preferred as signal collectors in BCIs, as they do not restrict users’ ambulation. The EEG evaluates the macroscopic dynamics of brain electrical activity via passive voltage measures on the scalp, with information characterized by low spatial resolution (of the order of centimeters) but high temporal resolution (of the order of milliseconds) [5], which is a great advantage for real-time BCI applications [6,7]. fNIRS is instead a diffuse optical imaging technique that estimates hemodynamic changes in oxy- (HbO) and deoxyhemoglobin (HbR) concentration, associated with the functional hyperemia following brain activity, and it is characterized by a lower temporal resolution than EEG due to the slow time course of hemodynamic fluctuations, and a higher spatial resolution (around 1 cm) [8,9].

In addition to fNIRS, diffuse optical imaging has been used to measure rapid changes in optical brain properties directly associated with neuronal activity. This method would allow for circumventing the restrictions imposed by the limited temporal resolution of fNIRS, while maintaining a high spatial resolution, by simply using the same technology employed for fNIRS but focusing on higher frequency ranges of the signal modulation. As demonstrated in vitro, the electrical activity of neurons is coupled with synchronous changes in Near Infrared (NIR) light scattering [10,11]. Moreover, changes in the intensity of NIR light transmitted through rat brain tissue have been observed in response to suprathreshold electrical stimulation [12]. In humans, Gratton and colleagues (1995) were the first to demonstrate that fast optical signals (FOS, also known as event-related optical signals, EROS), as assessed by means of modulation in light intensity or through changes in the average photons time of flight in the tissue, could detect localized brain activity with a temporal resolution of about 20 ms [13]. Notably, a proper FOS recording requires certain precautions. In the first place, temporal and spatial sampling could play a crucial role because signals are localized in both time and space. Moreover, FOS are characterized by a low signal-to-noise ratio (SNR) [14], which could be overcome by measuring a great number of trials and then calculating average responses. To enhance FOS’ detectability, multiple data analysis strategies have been proposed such as spectrum analysis [14], Independent Component Analysis (ICA) [15,16], and Generalized Linear Model (GLM) analysis [17]. FOS are typically recorded using longer (above 800 nm) wavelengths in the NIR spectrum because of the lower absorption characteristics of longer wavelengths, resulting in a higher SNR [18]. Studies employing a broad spectrum of NIR wavelengths show that longer wavelengths are more sensitive to FOS [12]. FOS has been measured during a variety of event-related tasks involving sensory, motor, auditory, and prefrontal cortices [13,18,19], as well as visual tasks [20]. Regarding the latter, FOS measured over the visual cortex can be used to create retinotopic maps [17].

**Figure 1:**
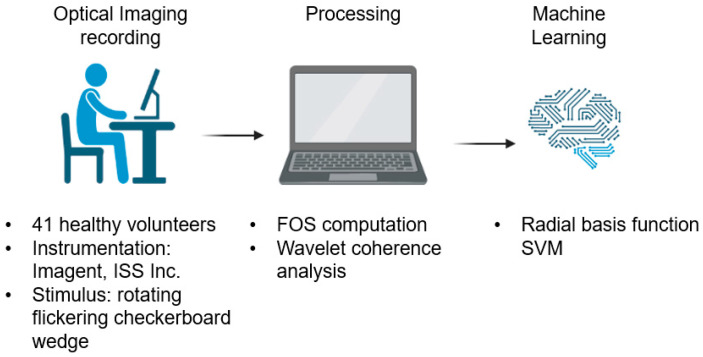
Schematic overview of the proposed method. The figure is created with BioRender.com (https://app.biorender.com/, accessed on: 26 April 2023).

The ability to estimate a retinotopic map shows the high temporal resolution of FOS, making this method ideally suited for BCI applications based on visual stimuli. FOS has rarely been used to feed data-driven machine learning (ML) approaches to classify cortical activation and deliver a successful classification of brain signals. In BCI systems, ML techniques play a dominant role in data analysis since they help with learning, comprehending, and interpreting complex brain activities [21]. Among the variety of classifiers available, the support vector machine (SVM) has been demonstrated to be highly effective for classifying brain signals [22,23,24,25,26,27]. Proulx et al. (2018), for example, used SVM and Linear Discriminant Analysis (LDA) using FOS (light intensity and phase delay, a measure of average photons time of flight using frequency-domain NIR systems) to distinguish between unusual and common responses [28]. The outcomes were combined using a weighted majority vote. FOS responses to rare and common images were classified among participants either offline, obtaining an average balanced accuracy of 62.5%, or online, with an average balanced accuracy of 63.6% [28]. The current study aimed to investigate the capability of combining FOS with SVM for single-trial retinotopy estimation in BCI applications. In particular, features computed through a wavelet coherence procedure applied to FOS were used as input for an SVM classifier in order to provide a classification of the visual quadrants relying on single-trial recordings. Figure 1 reports the block diagram of the proposed method.

## 2. Materials and Methods

This section describes participant enrollment and the experimental paradigm. In addition, a description of the optical instrumentation used to record FOS is provided, along with an explanation of the preprocessing of the signals and the ML analysis.

### 2.1. Participants

Forty-one healthy volunteers (age: 25–40 years, mean: 26 years, 18 males and 23 females) were enrolled in the experiment. Participants were right-handed, native English speakers, with normal or corrected to normal vision and hearing, and reported themselves in good health and free of medications known to affect the central nervous system. All participants were informed about the methodologies of the study and provided written informed consent. The study was approved by the University of Illinois at Urbana-Champaign Institutional Review Board, and it was performed according with the ethical standards of the Helsinki Declaration.

### 2.2. Visual Stimulation

The visual stimulus was a wedge of a reversing black and white checkerboard rotating counterclockwise around a gray fixation cross-located at the center of the screen. Participants were asked to focus on the fixation and silently count the number of times the fixation turned white. The wedge made a complete revolution in 48 s (1/48 Hz) and the checkerboard flickered at 5 Hz. Eight revolutions per subject were presented. Participants were sat comfortably in front of a computer screen located at a distance of 113 cm. Experiments were carried out in a dimly illuminated room.

### 2.3. Optical Imaging Recording

**Figure 2:**
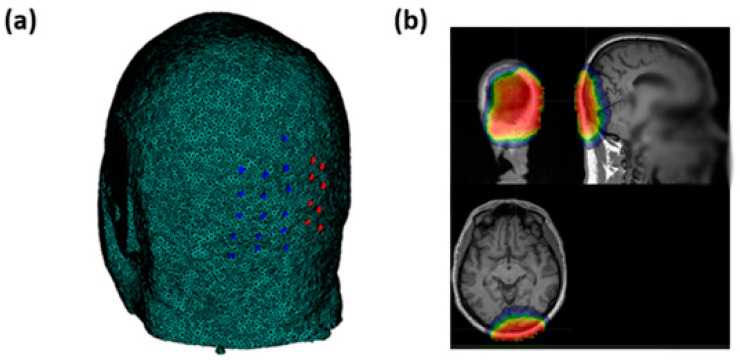
(**a**) Sources’ (blue circles) and detectors’ (red circles) locations on the occipital region. (**b**) Average sensitivity map obtained through the optical pad employed in an exemplary subject of the study.

Optical signals were recorded using an integrated set of 6 frequency-domain oximeters (Imagent, ISS Inc., 1602 Newton Dr, Champaign, IL, USA). The system consisted of 32 sources (16 laser diodes emitting at 690 nm and 16 emitting at 830 nm) and 16 Photomultiplier Tube (PMT) detectors, 8 of which were used for the current study. The light emitted by the sources was time-multiplexed and modulated at 110 MHz. To generate a 6.25 kHz heterodyne detection, the current supplying the PMTs was modulated at 110.00625 MHz. In addition, a Fast Fourier Transform (FFT) was applied to the 50 kHz sampled output current of the PMTs. Transformation to frequency domain enabled the recording of the average signal intensity (Direct Current, DC), modulated signal intensity (Alternating Current, AC), and phase delay (PH). Because of multiplexing across sources, the effective sampling rate for each channel (source–detector combination) was set at 39.0625 Hz, which is suitable for FOS recording [29]. To assess FOS responses to visual stimulation, we measured optical signals from the occipital region (centered around the Oz electrode location based on the standard 10–20 EEG system) using a custom helmet consisting of 16 source fibers (each source fiber composed of two sources at 690 nm and 830 nm of wavelength) and 16 detectors (Figure 2a). The optodes were arranged to create 256 overlapping channels (128 per wavelength) at different source–detector distances ranging from 20 to 70 mm. This montage allowed for different depth sensitivities covering the visual cortex. The average sensitivity map is reported in Figure 2b in a representative subject.

### 2.4. Fast Optical Signal Preprocessing

We used DC intensity and phase signals at the two wavelengths recorded and measured continuously over recording blocks lasting 6.26 min, beginning 1 s before the stimulation started, and lasting 1 s after the stimulation ended. For all signals, a zero-phase distortion FIR digital high-pass filter (2 Hz cutoff frequency) was applied to whole-block data. The high-pass cutoff frequency allowed for the elimination of slow drifts, and hemodynamic effects, and significantly reduced cardiac artifacts. Changes in phase signal expressed in degrees (FOS_PH_) were converted into changes in the average time of flight of photons using the following equation.

(1) $$\Delta t=\Delta \mathrm{PH}\cdot \pi /\left(360\pi \cdot \mathrm{Modulation}\right)$$

where Modulation is the frequency of light modulation (110 MHz). Changes in signal DC intensity were evaluated by converting the signals in optical densities (FOS_DC_) using the following equation:

(2) FOSDC=log(DCDCaveraged over block)

where DC_averaged over block_ is the average value of DC across the whole block. Signals recorded from all available channels were fed to the classifier.

### 2.5. Machine Learning Approach

For both the DC and phase components at the two wavelengths, feature extraction was performed by exploiting the temporal locking of the signals in the different optical channels during the response to the stimulus. Specifically, the average (over time and frequency) of the modulus of the wavelet coherence between the single channel and the average response among all channels was computed for each channel. This analysis was performed on individual trials in an integration time window of 512 ms (i.e., 20 sampling points) maximizing the overlap between the wavelet Cone Of Influence (COI) and the concatenated responses of two trials of 400 ms length [30]. 

The label associated with each 512 ms signal was assigned depending on the position of the rotating flickering checkerboard wedge on the screen, which represented illumination of a quadrant of the participants’ visual field since they were fixating the center of the wedge rotation. The label ‘top’ or ‘bottom’ was assigned if the center of the wedge was, respectively, between −45° and 45° quadrant or between 135° and 225° quadrant (with a counterclockwise rotation) (these angles were relative to the vertical meridian of the screen/visual field). The label ‘right’ or ‘left’ was assigned if the center of the wedge was, respectively, between 45° and 135° quadrant or between 225° and 315° quadrant.

All trials from all subjects were concatenated; hence, the SVM input matrix dimensions were 19,680 (number of trials in a single quadrant considering all subjects × 2 quadrants) × 128 (number of channels). The data were normalized (z-scored) with a label of 0 and 1 for the two classes considered for the specific classification required (either top vs. bottom or right vs. left quadrants). The SVM algorithm employed was a non-linear classifier using a Radial Basis Function (RBF) kernel, with the exponent γ (hyperparameter of the model) set to a standard value for z-scored inputs of γ = 0.5. The machinery was evaluated using 5-fold cross-validation. To avoid overfitting effects in the test sets, the folds were constructed by gathering all the data from the same subject in the same fold.

To test the performance of the classifier, the confusion matrix of each approach was computed, delivering sensitivity, specificity, and accuracy of the classification. Moreover, a Receiver Operating Characteristic (ROC) analysis was implemented, and the Area Under the ROC curve (AUC) was evaluated. The statistical significance of the AUC was computed by estimating the null hypothesis distribution through random shuffling of the output vector (1 million iterations).

Finally, the information transfer rate (ITR), which is a general evaluation metric for BCI systems that measures the quantity of information conveyed by the output of the system, was computed from the accuracy and the method response time (512 ms) to evaluate the performance of the classification for BCI applications.

**Figure 3:**
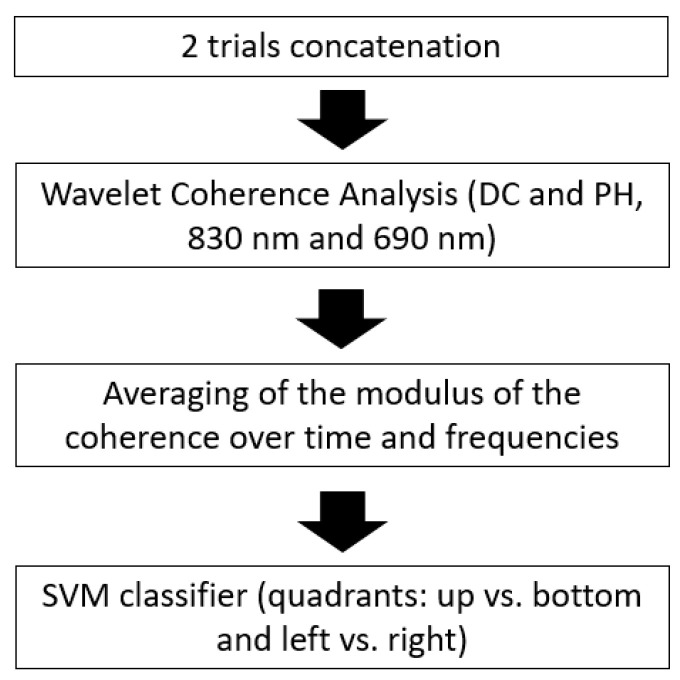
Flowchart describing the FOS processing pipeline for the visual quadrants’ classification.

The processing pipeline is described in the flowchart reported in Figure 3.

## 3. Results

**Figure 4:**
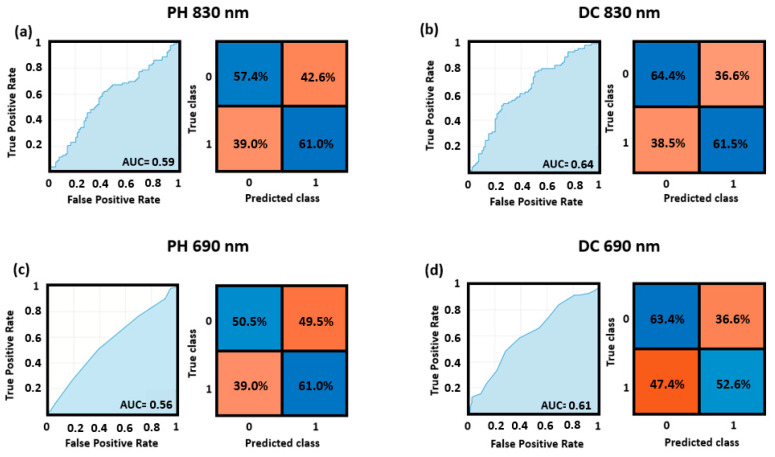
ROC curves and confusion matrices for the top vs. bottom quadrants’ classification obtained through SVM applied to (**a**) PH of the 830 nm wavelength, (**b**) DC of the 830 nm wavelength, (**c**) PH of the 690 nm wavelength, and (**d**) DC of the 690 nm wavelength.

Figure 4 reports the ROC curves and the confusion matrices for the optimized cutoff point obtained for the cross-validated classification performance of the top and bottom quadrants, for both the DC and PH at 830 nm and 690 nm wavelengths. The higher AUC (AUC = 0.64) was obtained by the model fed using the features computed from the DC at 830 nm, whereas the lower AUC was obtained by the model taking as input the features from the PH at 690 nm (AUC = 0.56). Note that all these values refer to the whole sample of 41 participants.

**Figure 5:**
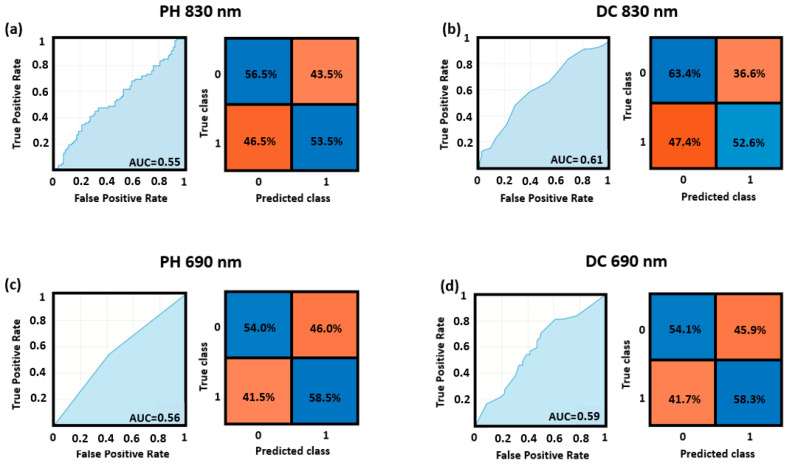
ROC curves and confusion matrices for the left vs. right quadrants’ classification obtained through SVM applied to (**a**) PH of the 830 nm wavelength, (**b**) DC of the 830 nm wavelength, (**c**) PH of the 690 nm wavelength, and (**d**) DC of the 690 nm wavelength.

Figure 5 reports the ROC curves and the confusion matrices for the best cutoff point delivered by the cross-validated classification of the right and left quadrants for the DC and PH at 830 nm and 690 nm wavelengths. The results refer to the whole study sample. The model fed with the features computed from the DC at 830 nm yielded the highest AUC (0.64), while the model fed with the features computed from the PH at 830 nm yielded the lowest AUC (0.55).

Table 1 reports all classification accuracies (100× number of correctly classified trials/total number of trials) obtained and the associated ITR. Note that almost all classifications were significantly better than chance (above 50%, *p* < 0.05), with two having a tendency towards significance (*p* < 0.1). The best classification was obtained for top vs. bottom quadrant classification assessed using the DC intensity using 830 nm light. The classification accuracy was qualitatively greater for DC intensity than for phase measures (58.79% vs. 56.55%, respectively), 830 nm than 690 nm light (58.79% vs. 56.55%, respectively), and for top–bottom than left–right discrimination (58.97% vs. 56.36%, respectively). The advantages for DC intensity and 830 nm light may both correspond to a higher signal-to-noise ratio for these measures, and was therefore predictable.

**Figure 6:**
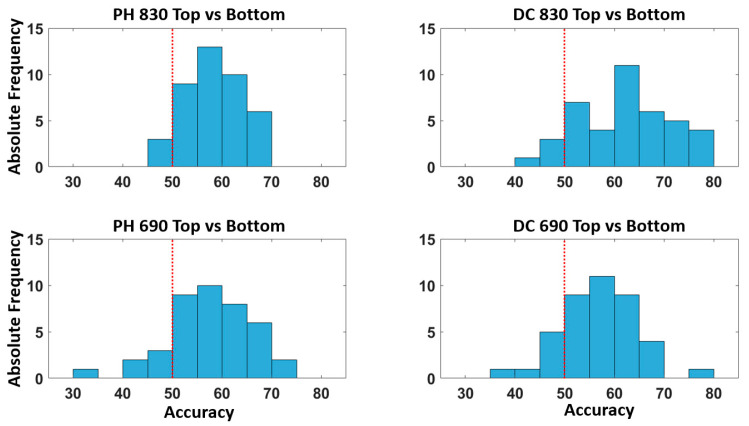
Distribution of the accuracy (%) across all the participants for the classification of the top and bottom quadrants. The red dashed line represents the above chance threshold.

Figure 6 describes the distribution of accuracies across all the participants for the classification of the top vs. bottom quadrants for all the optical signals considered. The figure shows that the model provides an above chance classification accuracy for the majority of the participants.

**Figure 7:**
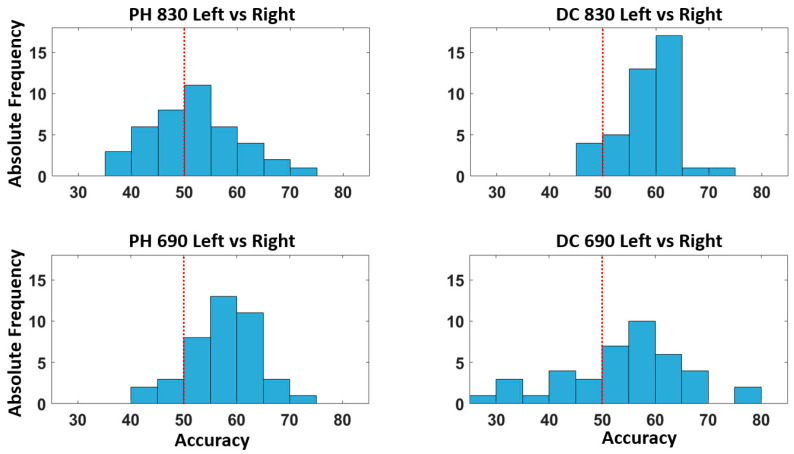
Distribution of the accuracy across all the participants for the classification of the left vs. right quadrants. The red dashed line represents the above chance threshold.

Figure 7 depicts the accuracy distribution associated with the left vs. right quadrants’ classification for each optical signal taken into consideration. Additionally in this case, the model provides a classification accuracy above chance for most of the participants.

Notably, the processing computation time provided by the proposed pipeline analysis was only 0.071 ± 0.007 s.

## 4. Discussion

FOS can monitor cortical activity with high temporal and spatial resolutions. Differently from fNIRS, FOS does not measure the slow hemodynamic response following brain activation but directly measures the fast changes in optical properties that are synchronous with neuron depolarization. Hence, FOS might provide higher ITR than fNIRS for real-time BCI applications. However, FOS are characterized by a lower SNR with respect to fNIRS requiring appropriate data analysis strategies to enhance their performance in BCI.

In this study, a retinotopy FOS-based framework was investigated for BCI implementations. Fast modulations in DC light intensity and phase shift at both 690 nm and 830 nm wavelengths were analyzed. The framework included a feature extraction algorithm based on the evaluation of the wavelet coherence of the signal in each channel (all over the visual cortex) with the average signal among channels. This approach highlighted the signal synchronization among channels soon after the stimulus. The average amplitude across time and frequency of the wavelet coherence was used to feed a cross-validated (5-fold) SVM classifier. The classifier was trained and tested to distinguish between the top and bottom visual stimulation quadrants, as well as the left and right stimulation quadrants. We demonstrated the feasibility of employing FOS for retinotopy mapping in BCI, obtaining classification accuracies above chance. The 830 nm wavelength FOS feature set produced significantly better classification results than the 690 nm wavelength [12,31]. Consistently with our findings, Lee and Kim (2010) demonstrated that FOS are more sensitive to neural activity when using light with a longer wavelength [12]. This finding is related to the increased spectral absorption at a shorter wavelength, which results in fewer photons reaching the detector and a lower SNR [32]. Notably, the wavelength-dependent classification outcomes imply that the findings are not associated with motion artifacts, as motion artifacts would have affected the paired-wavelength sources in the same way.

Interestingly, the performance of intensity data was found to be superior to that of phase. A previous study reporting on the FOS identification for BCI applications identified that the classification relying on DC intensity signals performed better than classifiers based on phase delay. These results are also in line with previous studies reporting the ability to detect FOS using DC signals but not using phase [33,34]. Moreover, using Monte Carlo simulations, Franceschini et al. demonstrated that changes in scattering caused smaller changes in phase delay than in intensity, which explains why phase delay has a lower SNR than intensity [35]. However, it should be mentioned that previous FOS investigations [31,36] demonstrated that phase delay is more accurate than DC for detecting FOS, and it was hypothesized that external noise sources, such as ambient light, could influence the FOS DC result [37]. However, the use of a 2 Hz high-pass filter in our study may help suppress some of these problems and improve the signal-to-noise ratio of the DC intensity signals.

We hypothesize that the lower classification performance of our phase data might be attributed to the variability in the locations of the optodes with respect to the brains across participants. Phase delay signals have larger gradients of spatial sensitivities and, consequently, are more affected by modifications in the channel locations [29]. This issue may be overcome by aligning optical channels with brain anatomy using subject-specific structural images (e.g., based on anatomical magnetic resonance images), reconstructing voxel-space single-trial data through inversion algorithms [38]. However, to support the use of DC data for FOS detection, it should be highlighted that systems able to produce a quick classification of cortical states without a precise co-registration with anatomy are advantageous for BCI.

Classification accuracy was also greater for top–bottom vs. left–right classification. This contrasts with the fact that the distance between regions of the primary visual cortex associated with the left and right visual fields is more distant than that associated with the top and bottom areas of the visual field. At first glance, this should make left–right discrimination easier than top–bottom discrimination. It is to be noted, however, that the optical montage used in the current study was based largely on optodes oriented in the left–right direction rather than the top–bottom direction over the occipital region. This arrangement meant that different channels covered higher and lower occipital regions (corresponding to the top–bottom dimension of the screen/visual field). At the same time, most channels collected data from relatively different regions in the horizontal (left–right) axis (corresponding to the left–right dimension of the screen/visual field). This may have translated into a different spatial resolution of the measure along the two axes. To test whether this is the case, the experiment should be re-run using montages with different dominant orientations. In other words, specific montages may generate quite different power levels for discriminating between brain activities, depending on how the individual channels overlap with the critical brain regions involved.

It should be noted that the average classification accuracy did not reach the threshold of 70%, which is considered the lower bound of accuracy for effective BCI communication [39]. However, in this study, single trials for FOS were considered without averaging, representing an improvement when compared to previous FOS studies, where at least 15 trials were averaged for FOS detection [28]. Moreover, Figure 6 clearly shows that a substantial proportion of participants (>20%) achieved a classification accuracy of >70% for the top–bottom quadrants’ classification. Moreover, for both the top–bottom and left–right classifications, the vast majority (>90%) of the participants had better than chance classifications.

Accurate signal augmentation, signal categorization, and noise reduction may be implemented to improve the performance of the method. The signal integrity can be improved by selecting the optimal light wavelengths and improving light injection and detection chains [40], whereas information can be enhanced by combining optical signals with other fast brain monitoring modalities, such as EEG [41,42]. Importantly, optical systems are ideal for multimodal acquisitions since they generally do not interfere with magnetic or electrical fields.

Notably, physiological noise may impair brain state classification, causing both false negatives and false positives [43]; however, FOS should be less affected by physiological contamination than fNIRS, simply because the FOS spectral bandwidth is at a higher frequency range than most physiological processes. Nonetheless, advanced signal processing might further mitigate physiological noise. Importantly, the diffuse optical tomography configuration utilized in this study offers multiple inter-optode distances, which would result in varying penetration depths delivering the ability to isolate the effect on deep brain layers decoupling them from scalp layers [44].

In addition, it should be noted that the classification performance in this study was assessed across participants. This practice has the advantage of not requiring training the classifier on each individual, greatly improving the ease of application of the BCI system. Indeed, the across-participant approach tends to reduce the reachable classification accuracy because of the physiological and experimental variability among different individuals [45]. The performances of BCI systems, particularly using EEG, are often assessed on a single-subject basis to account for inter-subject variability. Preliminary results on the dataset employed in this study demonstrated that single-subject and across-subject classifications were not statistically different; hence, the second approach was preferred to increase the generalization performance of the proposed model. The lower than expected single-subject classification was probably caused by the limited samples for each subject impairing the training of the classifier. Experiments using greater numbers of trials should be performed to evaluate the single-subject classification performance of FOS-based retinotopy. Indeed, ML frameworks improve their performance with larger datasets. Importantly, in this study, a nested cross-validation (nCV) was employed to protect the results against overfitting and generalize the results. In particular, a 5-fold cross-validation was employed. In nCV, the data are partitioned into folds, and the model is trained iteratively and in a nested way on all but one fold. The inner loop, as opposed to the exterior loop, which estimates the model’s performance across iterations (test), determines the optimal hyperparameter (validation). Hence, the model described in this study was validated using data that were not used to train the model.

Despite the low SNR of FOS limiting the ITR, the originality of this study lies in the ability to classify mental states through FOS without trial averaging in a 512 ms time window (only two trials were considered for each classification). This short time window, providing a certain level of classification accuracy, contributes to increasing the ITR, which is a crucial parameter for real-time human–machine interaction in BCI. Moreover, the average inference time of the proposed method is 0.071 s, thus making the classification suitable for real-world scenarios.

This result could encourage the use of optical signals in real-time BCI applications. Finally, more complex ML techniques investigating high-order non-linearities on larger data samples, such as deep learning, could be utilized to improve classification performance.

## 5. Conclusions

This study reports on a retinotopy frequency-domain FOS-based method for BCI applications. The method was tested across subjects by cross-validation. The procedure, exploiting the locking of the signal modulations among optical channels all located on the visual cortex and implementing a data-driven machine learning approach for discrimination, delivered a beyond chance performance when classifying visual stimulation quadrants for all signals (intensity or phase) and NIR wavelengths considered (690 nm and 830 nm). The best classification performance of around 63% accuracy was obtained when discriminating between the superior and inferior quadrants using light intensity modulation at the longer wavelength. The classification performances were obtained using a response time of only 512 ms with the best performance information transfer rate of around 6 bits per min. Although an increase in accuracy would be ideal for effective BCI, this approach is indeed the first attempt to develop a model for a generalizable retinotopy classification based on FOS without averaging the signal across several trials, highlighting the potentialities of FOS for real-time BCI.

## Figures and Tables

**Table 1:** Accuracies and ITR obtained by the SVM in classifying the quadrants from the different optical metrics.

Contrast	Optical Metric	Accuracy (%)	*p*-Value	ITR (bpm)
Top vs. bottom	PH 830	59.20	0.005	2.82
Top vs. bottom	DC 830	62.95	0.001	5.92
Top vs. bottom	PH 690	55.75	0.071	1.25
Top vs. bottom	DC 690	58.00	0.021	2.23
Left vs. right	PH 830	55.00	0.082	0.87
Left vs. right	DC 830	58.00	0.016	2.23
Left vs. right	PH 690	56.25	0.037	1.70
Left vs. right	DC 690	56.20	0.039	1.70

## Data Availability

The data are available on request to the corresponding author.

## Nomenclature

AC	Alternating Current light intensity
AUC	Area Under the Curve
BCI	Brain–computer interface
DC	Direct Current light intensity
EEG	Electroencephalography
fNIRS	Functional near-infrared spectroscopy
FOS	Fast optical signals
ITR	Information transfer rate
ML	Machine learning
PH	Phase delay
RBF	Radial Basis Function
ROC	Receiver Operating Characteristic
SNR	Signal-to-noise ratio
SVM	Support vector machine
